# Iron deficiency, independently of anaemia, is associated with decreased cardiac systolic function and alteration of myocardial mitochondrial metabolism in a mouse model

**DOI:** 10.1186/2197-425X-3-S1-A979

**Published:** 2015-10-01

**Authors:** T Gaillard, E Rineau, N Gueguen, F Prunier, D Henrion, S Lasocki

**Affiliations:** Anesthésie Réanimation, Univ. Hospital Angers, Angers, France; CNRS 6214 - INSERM 1083, Univ. Hospital Angers, Angers, France; UPRES EA 3860, Univ. Hospital Angers, Angers, France

## Introduction

Iron deficiency (ID) is very frequent in cardiac patients and has been associated with altered cardiac function. The correction of ID in patients with heart failure improves clinical symptoms, in patients with or without anemia [1, 2]. Alteration of mitochondrial metabolism due to ID may be responsible for these observations.

## Objectives

We developed a mouse model of ID without anaemia to assess the link between ID and cardiac function, to characterize the mitochondrial metabolism and to assess the effect of iron treatment.

## Methods

4 groups of C57BL/6 mices were compared : ID (obtained by a first retro-orbital puncture followed by 3 weeks of low iron diet); control mices (CT group). These mice received either an intraperitoneal saline injection or a single dose of 15 mg/kg carboxymaltose ferric at week 1 (ID+I and CT+I groups). Effort capacity was measured using a Rotarod (ie by assessing the racing time) and a forced swimming test (a load equal to 5% of the body weight was attached at the base of the tail and the duration of swim was measured). Left Ventricular Ejection Fractions (LVEF) were measured using transthoracic echocardiography done by a cardiologist blinded to the iron status. After sacrifice at W3, haemoglobin (Hb) was measured and activities of the different mitochondrial respiratory chain complexes were measured by spectrophotometry on myocardial muscle. Myoglobin and complex I were quantified using BN-PAGE.

## Results

**Figure 1:**
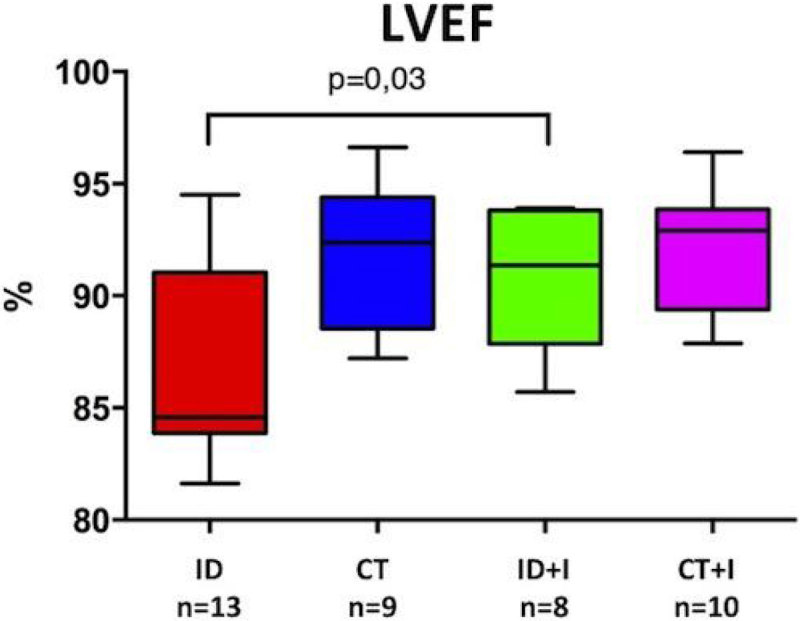
Figure 1

**Figure 2:**
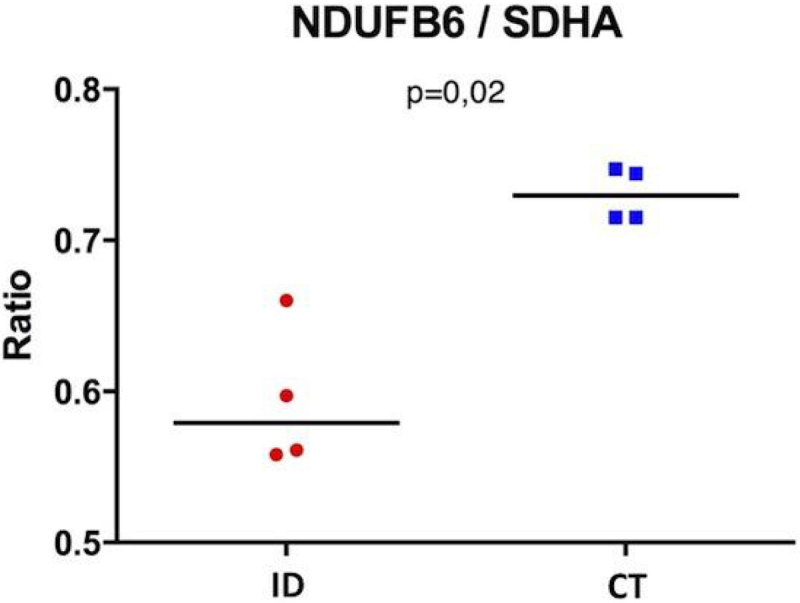
Figure 2

At W3, Hb level was not different between ID, CT and ID+I groups (13.9 [13.6 to 14.3] vs 14 [13.4 to 14.4] vs 14,8 [14.3 to 14.95] g/dL, p = 0.89). The racing time of ID mice were lower than CT mice (3.8 [3.4-5.2] vs 9.1 [5.7-11.5] min, p = 0.005) but swimming time was not different (8.2[4.3-10.4] vs 10.8[5.8-12.6] min, p = 0.06). ID+I mice had a longer swimming time than ID mice (13 [10.20 to 15.8] vs 9.1 [6,6 to 11,6] min. LVEF were significantly reduced in the ID group compared to CT, but not in ID+I (Figure 1). The complex I activity was reduced by 32% in ID vs CT mice (740 [507 to 1010] vs 1086 [819 to 1241] nmol/min/mg, p = 0.02), this was associated with a reduction in the amount of complex I in ID mitochondria (figure 2). Activities of the other mitochondrial complexes (II, III, IV) were not altered. Iron treatment was associated with a normal amount complex I compared to CT mice. There was no difference in myoglobin content between ID and CT groups.

## Conclusions

In this mice model of ID without anaemia, we observe early decrease in exercise capacity and LVEF, which could be linked to a decrease in mitochondrial complexe I activity and amount. All these observations were reversed by a single iron injection.
